# Development and validation of a nomogram for risk prediction of nephrolithiasis recurrence in patients with primary hyperparathyroidism

**DOI:** 10.3389/fendo.2022.947497

**Published:** 2022-08-31

**Authors:** Yihong Zhou, Xi Chu, Dong Jiang, Xiang Wu, Jiarong Xu, Hao Qi, Yuxin Tang, Yingbo Dai

**Affiliations:** ^1^ Department of Urology, The Fifth Affiliated Hospital of Sun Yat-sen University, Zhuhai, China; ^2^ Guangdong Provincial Key Laboratory of Biomedical Imaging, The Fifth Affiliated Hospital, Sun Yat-sen University, Zhuhai, China

**Keywords:** primary hyperparathyroidism, nephrolithiasis, recurrence, nomogram, risk prediction

## Abstract

**Background:**

Nephrolithiasis is a common complication of primary hyperparathyroidism (PHPT), and the recurrence of nephrolithiasis in patients with PHPT is also an urgent concern. What is worse, there is a scarcity of recommended evaluation to predict the risk of nephrolithiasis recurrence in patients with PHPT. This study was aimed to develop and validate a nomogram to facilitate risk assessment in patients with PHPT.

**Methods:**

A total of 197 patients with PHPT were retrospectively included in this study from September 2016 to August 2021. Patients’ demographic data, blood test parameters, urinalysis, stone parameters, and surgical intervention were collected. Extracted variables were submitted to a least absolute shrinkage and selection operator (LASSO) regression model. A nomogram was built and validated according to the area under the curve (AUC) value, calibration curve, and decision curve analysis.

**Results:**

According to the LASSO regression and logistic regression analyses, five predictors were derived from 22 variables: creatinine, uric acid, bilateral stone, multiplicity, and surgery. The AUC and concordance index of the training cohort and validation cohort were 0.829 and 0.856, and 0.827 and 0.877, respectively. The calibration curve analysis and the decision curve analysis showed that the nomogram had an adequate prediction accuracy.

**Conclusion:**

We built a useful nomogram model to predict the risk of nephrolithiasis recurrence in patients with PHPT. This would assist clinicians to provide appropriate advices and managements for these patients.

## Introduction

Primary hyperparathyroidism (PHPT) is the third most common endocrine disease after diabetes mellitus and thyroid disorders, with an incidence of approximately 65.5 and 24.7 individuals per 100,000 in women and men, respectively (1, 2). Generally, PHPT occurs in post-menopause women. However, no significant difference exists in the population under the age of 45 years (3). Parathyroid dysfunction is the main cause of PHPT, with excessive release of parathyroid hormone (PTH) and disorder of normal regulation. As a result of increased level of PTH, the homeostasis of calcium and phosphate is disrupted, which leads to hypercalcemia through increased renal tubular calcium reabsorption (4). While hypercalcemia is a contributing factor in stone formation, the kidney is a principal target of PHPT, resulting in nephrolithiasis.

It has been reported that about 10% of the patients had pre-existing nephrolithiasis at the time of diagnosis of PHPT (5). Moreover, the incidence of nephrolithiasis in patients with PHPT is four times that of the general population (6). In addition to the high incidence, the nephrolithiasis recurrence in patients with PHPT is also an urgent concern. Huang et al. followed a cohort of 1,252 patients with PHPT and found an overall nephrolithiasis recurrence rate of 31.3%. For patients under observation treatment, the nephrolithiasis recurrence rate was 13.7%, 22.3%, and 29.4% in the 5-, 10-, and 15-year follow-up, respectively (7). Some studies have suggested several factors for kidney stone recurrence, such as age, body mass index (BMI), and 24-h urine parameters (8–10). However, to our knowledge, there is a lack of recommended evaluation to predict the risk of nephrolithiasis recurrence in patients with PHPT.

The main objective of this study was therefore to identify the significant predictive factors for nephrolithiasis recurrence in patients with PHPT and to develop and validate a nomogram to facilitate risk assessment. This would assist clinicians to provide appropriate advices and managements for these patients.

## Methods

### Data sources and study subjects

This study was approved by the Ethics Committee of the Fifth Affiliated Hospital of Sun Yat-sen University (No. K73-1). Data were retrospectively obtained from the medical records between September 2016 and August 2021 at the Fifth Affiliated Hospital of Sun Yat-sen University. All patients were diagnosed as nephrolithiasis with PHTH. PHTH was defined as PTH level > 69 pg/ml with a normal or elevated albumin-adjusted calcium (11, 12). The exclusion criteria were as follows: (1) secondary hyperparathyroidism, such as chronic kidney disease, vitamin D deficiency, and gastrointestinal malabsorption; and (2) intake of medications such as diuretics, hydrochlorothiazide, and lithium.

### Variables collected and outcomes

Data on the following parameters were collected: sex, age, BMI, medical history (hypertension and diabetes mellitus), blood test parameters (corrected calcium, PTH, phosphorus, chlorine, potassium, triglycerides, high-density lipoprotein, low-density lipoprotein, alkaline phosphatase, creatinine, and uric acid), urinalysis (pH and crystal), stone parameters (bilateral stone, multiplicity, and stone composition), and intervention (ESWL, PCNL, and RIRS). Albumin-adjusted calcium level was calculated as follows: corrected calcium (mmol/L) = measured calcium (mmol/L) + (40 − serum albumin concentration (g/L) × 0.02).

Patients were followed up every 3–6 months, with a range of 3–64 months and a mean follow-up of 25 months. Recurrence of nephrolithiasis was the primary endpoint, which was defined as a new kidney stone on ultrasound and/or CT scan occurring more than 30 days after intervention.

### Statistical analysis

SPSS 19.0 (IMB SPSS, Inc., Chicago, IL, USA) and R software (R version 4.1.1, R Project, www.r-project.org) were used for data analysis. Parameters were described as mean ± SD. When comparing the baseline characteristics, chi-square test or Fisher’s exact test was performed for categorical variables and Wilcoxon signed-rank test for quantitative variables. Modeling time to recurrence of nephrolithiasis was performed using Kaplan–Meier survival analysis. A *p*-value < 0.05 was considered to be statistically significant.

The overall cohort was randomly assigned 70% of the patients to the training cohort and 30% to the validation cohort. Extracted variables were submitted to a least absolute shrinkage and selection operator (LASSO) regression model for data dimensionality reduction and feature selection. Then, independent risk factors associated with recurrence of nephrolithiasis were selected using the logistic regression analysis, and a nomogram was built in the training cohort. A receiver operating characteristic (ROC) curve was subsequently drawn on the basis of predictive factors, and the sensitivity and the specificity were evaluated using the area under the curve (AUC) value. The concordance index (C-index) was used to evaluate the discrimination ability of the nomogram. Calibration curve and decision curve analysis were performed to examine the performance characteristics and assess the clinical benefits. Finally, the validation cohort was used to validate the nomogram constructed in the training cohort.

## Results

### Patient characteristics

A total of 197 patients were included in the current study, with 105 men and 92 women. According to the ratio of 7:3, 140 patients were randomly assigned into the training cohort and 57 patients in the validation cohort. The clinical characteristics of patients in the training cohort and the validation cohort are summarized in **Table 1**. There was no difference in distribution of all characteristics between the training cohort and the validation cohort.

**Table 1:** Comparison of characteristics between the training and validation cohort.

Characteristic	All cases n = 197	Training cohort n = 140	Validation cohort n = 57	*p* value
Sex				0.845
Male	105	74	31	
Female	92	66	26	
Age (years)				0.109
<45	60	39	21	
45–65	109	84	25	
>65	28	17	11	
Hypertension				0.745
Yes	48	35	13	
No	149	105	44	
Diabetes mellitus				0.711
Yes	27	20	7	
No	170	120	50	
BMI (kg/m^2^)				0.360
<25	118	87	31	
25–30	60	42	18	
>30	19	11	8	
Corrected calcium (mmol/L)				0.241
≤2.52	167	116	51	
>2.52	30	24	6	
PTH (pg/ml)				0.703
≤90	125	90	35	
>90	72	50	22	
Phosphorus (mmol/L)				0.627
≤0.85	46	34	12	
>0.85	151	106	45	
Chlorine (mmol/L)				0.908
≤105	151	107	44	
>105	46	33	13	
Potassium (mmol/L)				0.723
≤4.0	86	60	26	
>4.0	111	80	31	
Triglycerides (mmol/L)				0.303
≤1.88	153	106	47	
>1.88	44	34	10	
HDL (mmol/L)				0.429
≤1.08	102	75	27	
>1.08	95	65	30	
LDL (mmol/L)				0.326
≤3.12	161	112	49	
>3.12	36	28	8	
ALP (U/L)				0.949
≤90	148	105	43	
>90	49	35	14	
Creatinine (mg/dl)				0.714
≤0.916	79	55	24	
>0.916	118	85	33	
Uric acid (μmol/L)				0.889
Normal	116	82	34	
Abnormal	81	58	23	
Urine pH				0.939
<6	40	29	11	
6	115	82	33	
>6	42	29	13	
Urine crystal				0.855
Positive	22	16	6	
Negative	175	124	51	
Bilateral				0.644
Yes	88	64	24	
No	109	76	33	
Multiple				0.703
Yes	125	90	35	
No	72	50	22	
Stone composition				0.218
Calcium oxalate	121	91	30	
Carbonate apatite	50	31	19	
Others	26	18	8	
Intervention				0.847
ESWL	24	18	6	
PCNL	88	61	27	
RIRS	85	61	24	

BMI, body mass index; PTH, parathyroid hormone; HDL, high-density lipoprotein; LDL, low-density lipoprotein; ALP, alkaline phosphatase; ESWL, extracorporeal shock wave lithotripsy; PCNL, percutaneous nephrolithotomy; RIRS, retrograde intrarenal surgery.

### Recurrence of nephrolithiasis

**Figure 1:**
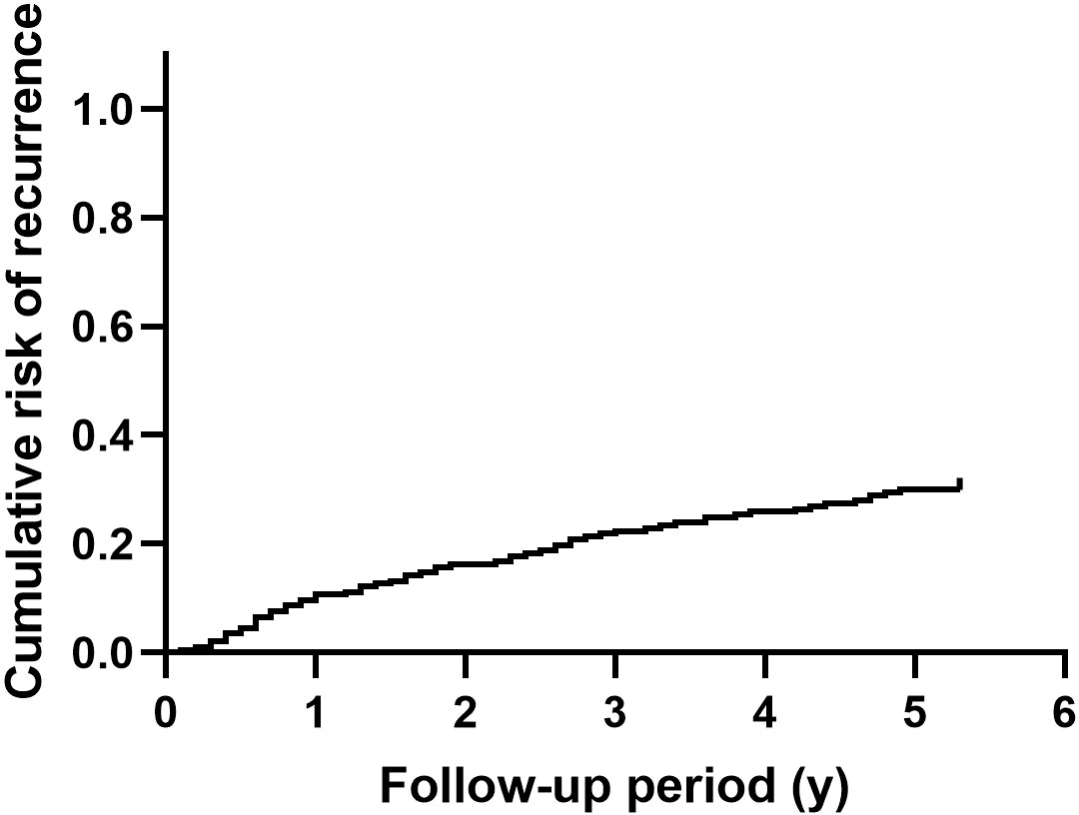
The cumulative recurrence rate of nephrolithiasis in patients with primary hyperparathyroidism.

After surgical intervention, 59 patients had at least one recurrent episode of nephrolithiasis. Overall recurrence rate of nephrolithiasis was 29.9%. **Figure 1** shows the cumulative recurrence rate of the entire cohort. Detailed characteristics of patients with or without nephrolithiasis recurrence are summarized in **Supplementary Table 1**.

### Independent predictors for nephrolithiasis recurrence

**Figure 2:**
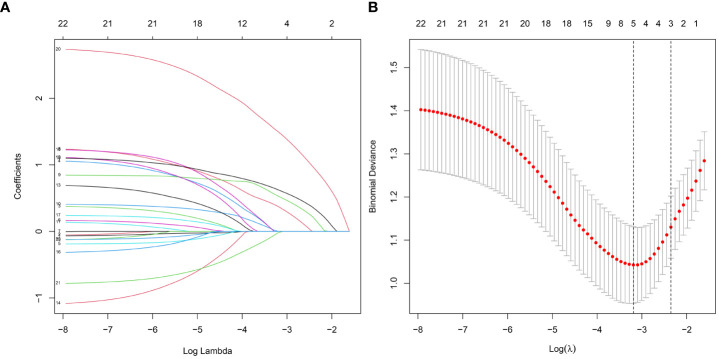
Features selection by LASSO. **(A)** Profiles of the LASSO coefficients for the 22 candidate variables. **(B)** Ten-fold cross-validation for tuning parameter selection in the LASSO model.

To screen out the independent predictors, all variables were preliminarily analyzed using LASSO analysis in the training cohort. Five potential predictors were derived from 22 variables (**Figure 2**). Finally, creatinine, uric acid, bilateral stone, multiplicity, and surgery were identified. **Table 2** shows the Odds ratio (OR) and 95% CI values of these predictors using the logistic regression analysis.

**Table 2:** Logistic regression analysis of independent risk factors associated with recurrence of nephrolithiasis in the training cohort.

Intercept and variable	β	Odds ratio (95% CI)	*p* value
Intercept	− 2.955	0.052 (0.009–0.218)	<0.001
Creatinine	0.940	2.560 (1.003–6.819)	0.053
Uric acid	0.909	2.483 (1.017–6.197)	0.047
Bilateral stone	1.063	2.895 (1.160–7.523)	0.025
Multiplicity	2.712	15.064 (3.669–92.151)	<0.001
Surgery PCNL RIRS	− 1.578 − 1.735	0.206 (0.032–1.136) 0.176 (0.029–0.936)	0.076 0.046

### Development and validation of the nomogram

**Figure 3:**
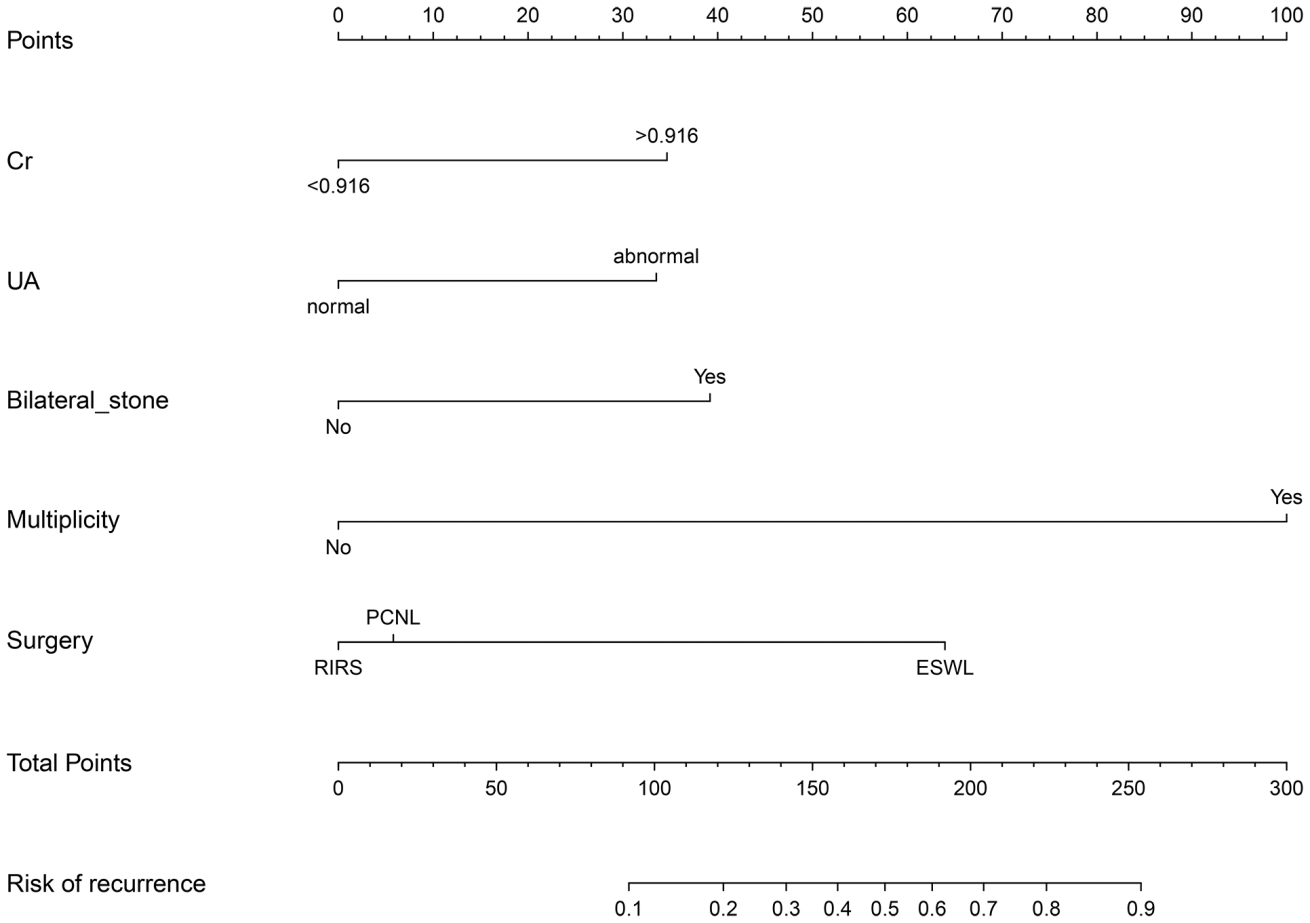
Nomogram predicting risk of nephrolithiasis recurrence in patients with primary hyperparathyroidism. Cr, creatinine (mg/dl); UA, uric acid (μmol/L); PCNL, percutaneous nephrolithotomy; RIRS, retrograde intrarenal surgery.

**Figure 4:**
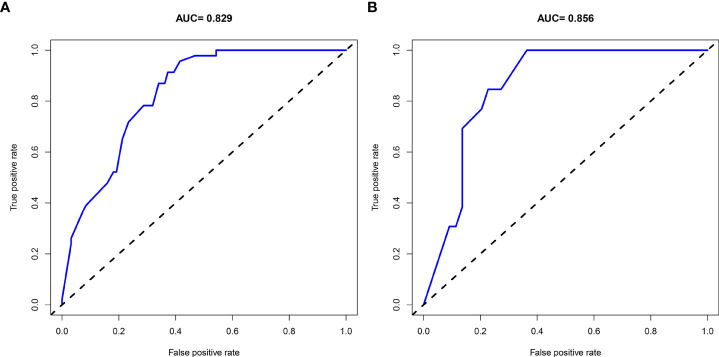
AUC of the ROC curve in training cohort **(A)** and validation cohort **(B)**.

A nomogram was then constructed on the basis of the abovementioned five predictive factors for nephrolithiasis recurrence in the training cohort (**Figure 3**). The nomogram indicated that multiplicity has the greatest influence on the nephrolithiasis recurrence of patients with PHPT, followed by surgery, bilateral stone, creatinine, and uric acid. The AUCs of the training cohort and validation cohort were 0.829 and 0.856, respectively (**Figure 4**). In addition, the C-index values of the training cohort and validation cohort were 0.827 and 0.877, respectively. Both the AUC and C-index value indicated that this nomogram had medium prediction accuracy. Moreover, this nomogram also showed adequate prediction accuracy on the basis of the calibration curve analysis (**Supplementary Figure 1**). Furthermore, the net benefit was comparable with several overlaps on the basis of the nephrolithiasis recurrence risk nomogram according to the decision curve analysis (**Supplementary Figure 2**).

## Discussion

Nephrolithiasis is a common disease in the general population, with an estimated prevalence of 10%–15% (13, 14). This condition is a considerable economic burden, costing the healthcare system more than $10 billion per year (15). In addition, a 50% risk of nephrolithiasis recurrence is observed at 7–10 years in the absence of specific treatment (10, 16). Whereas for patients after surgical treatment, 17%–21% had recurrent nephrolithiasis requiring a repeat surgery (17, 18). For patients treated with percutaneous nephrolithotomy (PCNL), the recurrence rate can be even as high as 40% (19). In addition, when with certain comorbidities, the risk of nephrolithiasis recurrence will increase. For example, 23% patients with type 2 diabetes will have a stone recurrence, and the recurrence rate in patients with metabolic syndrome is 3.2-fold higher compared with those in the control group (19, 20).

PHPT is a common endocrine disease characterized by hypercalcemia, which leads to a high incidence and recurrence rate of nephrolithiasis. Mollerup and Lindewald found that the recurrence rate of nephrolithiasis within 5 years was 30% in patients with PHPT (21). Similarly, in a cohort of 1,252 patients with PHPT, the overall recurrence rate of nephrolithiasis was 31.3% during an average follow-up of 8 years (7). In the current study, the overall recurrence rate of nephrolithiasis was 29.9%, which was consistent with the above studies.

Although with high recurrence rate of nephrolithiasis in patients with PHPT, no prediction model for nephrolithiasis recurrence in patients with PHPT has been reported to date. In the present study, a nomogram for risk prediction of nephrolithiasis recurrence in patients with PHPT was developed and validated. The final model was based on five predictors: creatinine, uric acid, bilateral stone, multiplicity, and surgery. The AUC and C-index of the training cohort and validation cohort were 0.829 and 0.856, and 0.827 and 0.877, respectively. Both the AUC and C-index value suggested that the nomogram could provide good prediction. This model would be helpful to provide appropriate advices and managements for patients with PHPT.

Stone burden is widely used for determining the management of urolithiasis. For kidney stones, evaluation of stone burden has shown to be a predictor of the clinical outcome of different surgical treatments, including flexible ureteroscopy and PCNL (22, 23). Recently, stone burden has also been proved to be associated with the nephrolithiasis recurrence (24, 25). Stone multiplicity, stone size, total stone volume, and bilateral stones are used to quantify stone burden. Observational and retrospective studies showed that the number of stones detected on imaging are associated with higher rates of recurrence (9, 26). Moreover, bilateral stones are also predictive of nephrolithiasis recurrence. Selby and colleagues found that patients with bilateral stones had a 1.8-fold increase in risk for encountering future symptomatic stone events (27). The current model indicated that stone multiplicity has the greatest influence on the nephrolithiasis recurrence of patients with PHPT (OR = 15.064, 95% CI: 3.669–92.151, *p* < 0.001). In addition, bilateral stone was also one of the predictive factors (OR = 2.895, 95% CI: 1.160–7.523, *p* = 0.025). These findings indicate that stone burden is a key predictor for nephrolithiasis recurrence of patients with PHPT.

Extracorporeal shock wave lithotripsy (ESWL), PCNL, and retrograde intrarenal surgery (RIRS) are the three major treatments for nephrolithiasis. With simple anesthesia requirement and being an outpatient surgery, ESWL is a choice with better patient acceptance. However, a lower stone free rates in patients after ESWL was identified when the effectiveness was investigated (28, 29). Several studies also compared the rate of stone recurrence between ESWL and PCNL, RIRS. During an average follow-up period of 19.2 years, Amy et al. found that ESWL had a higher stone recurrence than PCNL (53.3% vs. 36.8%, P = 0.033) (30). Similarly, increased probability of stone recurrence after ESWL was found when compared with RIRS (35.4% vs. 17.2%, P = 0.009) (31). In the current study, we found surgical intervention was a predictive factor for nephrolithiasis recurrence in patients with PHPT. Patients with ESWL would encounter a higher rate of stone recurrence compared with those treated with PCNL and RIRS.

Uric acid is the end product of purine metabolism, and high level of serum uric acid (SUA) is associated with several health disorders, including diabetes, cardiovascular diseases, metabolic syndrome, and cancer. A clear association has been identified between SUA and nephrolithiasis (32). PTH could decrease the secretion of uric acid in the renal tubular, leading to an increase of SUA. Recently, studies showed that patients with PHPT had a significantly higher level of SUA compared with those without PHPT (33). In this study, our results indicated that high level of SUA increased the rate of nephrolithiasis recurrence. Interestingly, outcomes of randomized controlled trials showed that SUA-lowering treatment decreased the risk of nephrolithiasis recurrence (34). Thus, controlling the level of SUA may be a potential measure to reduce stone recurrence in patients with PHPT.

There are certain limitations in the present study. First, although with a relatively good prediction accuracy, our study was retrospectively designed in a single center and lacked an external population to validate the predictive model. Second, studies have showed some potential variables that are related to nephrolithiasis recurrence, such as 24-h urine collection and parathyroidectomy. The results of existing research studies are contradictory (7, 35–37), and we tried to elucidate their roles in nephrolithiasis recurrence in patients with PHPT. However, these variables were not included due to the small number. To provide a more convinced result, a multi-center and large sample cohorts need be performed in the future.

## Conclusion

In conclusion, we built a useful nomogram model to predict the risk of nephrolithiasis recurrence in patients with PHPT. This would assist clinicians to provide appropriate advices and managements for these patients.

## Data availability statement

The original contributions presented in the study are included in the article/**Supplementary Material**. Further inquiries can be directed to the corresponding authors.

## Ethics statement

The studies involving human participants were reviewed and approved by the Ethics Committee of the fifth affiliated hospital of Sun Yat-sen University (No. K73-1). The patients/participants provided their written informed consent to participate in this study.

## Author contributions

YZ, YT, and YD designed the concept of the study. YZ, XC, and DJ collected and analyzed the data. XW, JX, and HQ critically revised the analysis. YZ wrote the draft manuscript. All authors listed have made a substantial, direct, and intellectual contribution to the work and approved it for publication.

## Funding

This study was supported by the Guangdong Medical Research Foundation (No. A2021358 to YZ) and the Natural Science Foundation of Guangdong Province (No. 2019A1515012116 to YD).

## Conflict of interest

The authors declare that the research was conducted in the absence of any commercial or financial relationships that could be construed as a potential conflict of interest.

## Publisher’s note

All claims expressed in this article are solely those of the authors and do not necessarily represent those of their affiliated organizations, or those of the publisher, the editors and the reviewers. Any product that may be evaluated in this article, or claim that may be made by its manufacturer, is not guaranteed or endorsed by the publisher.
