# Supplementary figures and images for: Development and validation of a nomogram for risk prediction of nephrolithiasis recurrence in patients with primary hyperparathyroidism
Source: Front Endocrinol (Lausanne). 2022 Aug 31;13:947497. doi: 10.3389/fendo.2022.947497 (PMC9470877; doi:10.3389/fendo.2022.947497)

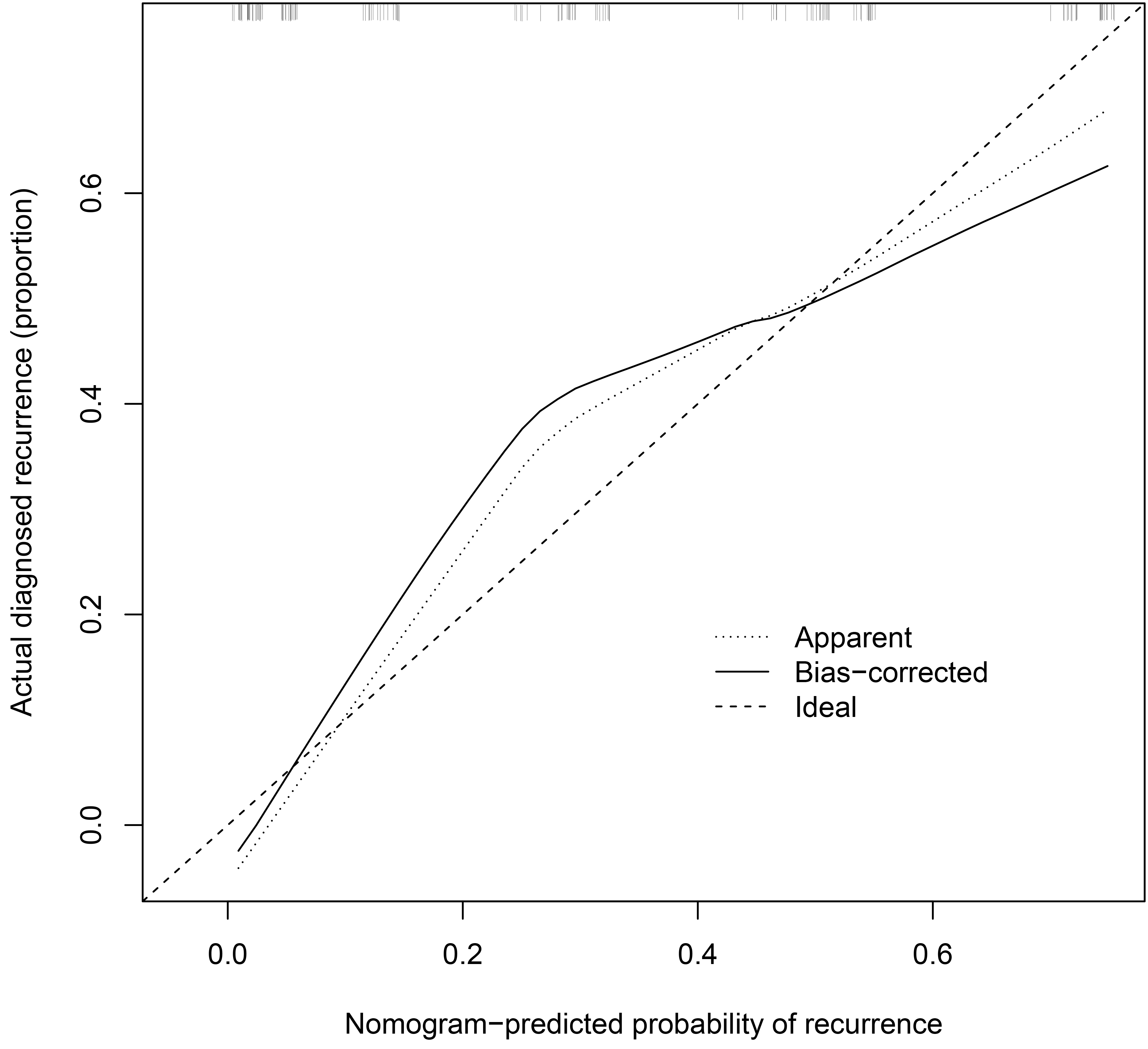

Supplement: Supplementary file 1 [file Image_1.tif]

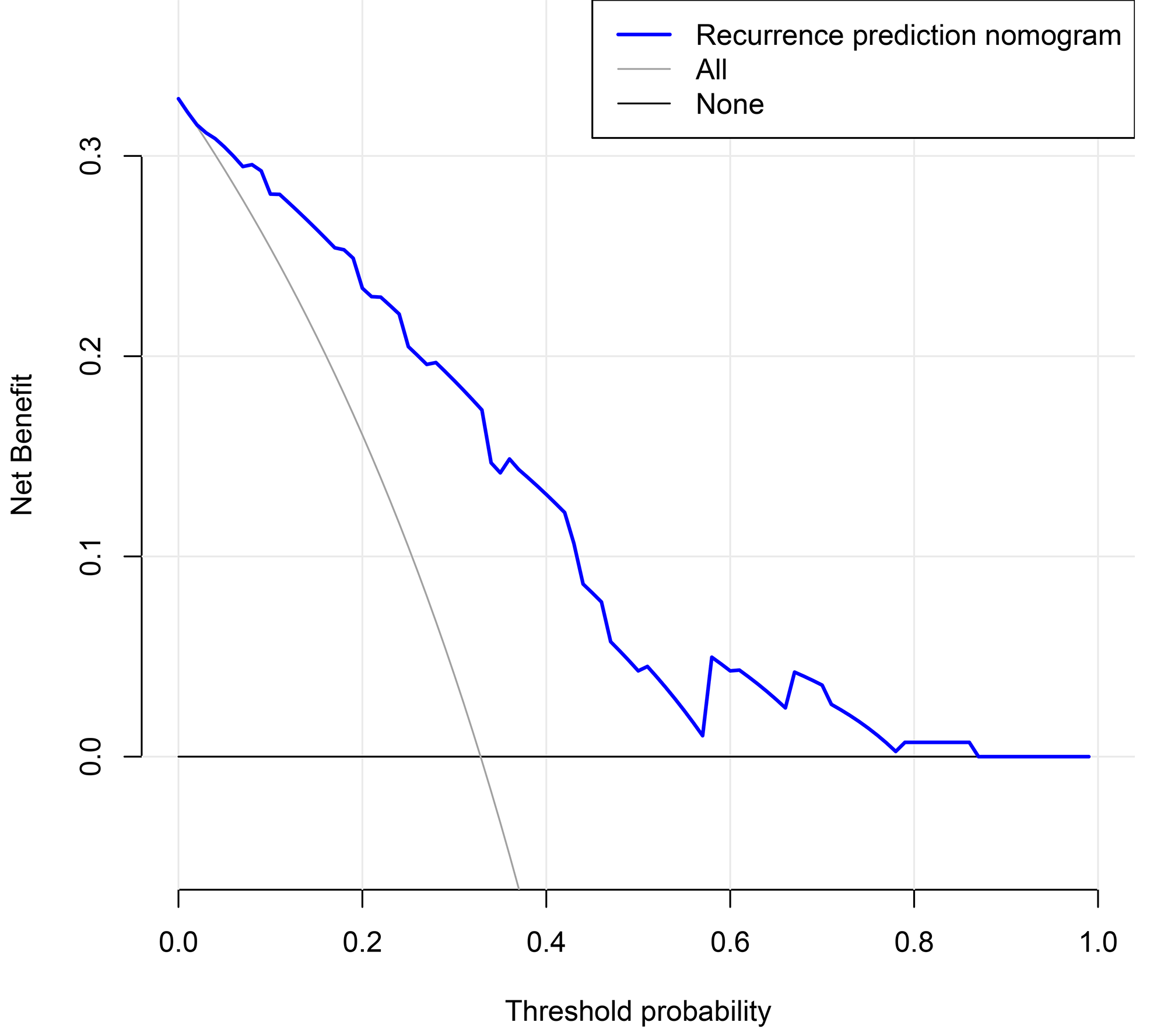

Supplement: Supplementary file 2 [file Image_2.tif]
