# Supplementary material for: Development and validation of a nomogram for risk prediction of nephrolithiasis recurrence in patients with primary hyperparathyroidism
Source: Front Endocrinol (Lausanne). 2022 Aug 31;13:947497. doi: 10.3389/fendo.2022.947497 (PMC9470877; doi:10.3389/fendo.2022.947497)
Supplement: Supplementary file 3 [file Table_1.docx]

Supplementary Table 1. Comparison of characteristics between recurrence group and no recurrence group.

| Characteristic | All cases  n = 197 | Recurrence  n = 59 | No recurrence  n = 138 | *p* value |
| --- | --- | --- | --- | --- |
| Sex |  |  |  | 0.166 |
| Male | 105 | 27 | 78 |  |
| Female | 92 | 32 | 60 |  |
| Age (years) |  |  |  | 0.071 |
| < 45 | 60 | 12 | 48 |  |
| 45 - 65 | 109 | 35 | 74 |  |
| > 65 | 28 | 12 | 16 |  |
| Hypertension |  |  |  | 0.821 |
| Yes | 48 | 15 | 33 |  |
| No | 149 | 44 | 105 |  |
| Diabetes mellitus |  |  |  | 0.679 |
| Yes | 27 | 9 | 18 |  |
| No | 170 | 50 | 120 |  |
| BMI (kg/m^2^) |  |  |  | 0.052 |
| < 25 | 118 | 43 | 75 |  |
| 25-30 | 60 | 12 | 48 |  |
| > 30 | 19 | 4 | 15 |  |
| Corrected calcium (mmol/L) |  |  |  | 0.192 |
| ≤2.52 | 167 | 47 | 120 |  |
| > 2.52 | 30 | 12 | 18 |  |
| PTH (pg/mL) |  |  |  | 0.152 |
| ≤90 | 125 | 33 | 92 |  |
| > 90 | 72 | 26 | 46 |  |
| Phosphorus (mmol/L) |  |  |  | 0.055 |
| ≤0.85 | 46 | 19 | 27 |  |
| > 0.85 | 151 | 40 | 111 |  |
| Chlorine (mmol/L) |  |  |  | 0.120 |
| ≤105 | 151 | 41 | 110 |  |
| > 105 | 46 | 18 | 28 |  |
| Potassium (mmol/L) |  |  |  | 0.387 |
| ≤4.0 | 86 | 23 | 63 |  |
| > 4.0 | 111 | 36 | 75 |  |
| Triglycerides (mmol/L) |  |  |  | 0.660 |
| ≤1.88 | 153 | 47 | 106 |  |
| > 1.88 | 44 | 12 | 32 |  |
| HDL (mmol/L) |  |  |  | 0.157 |
| ≤1.08 | 102 | 26 | 76 |  |
| > 1.08 | 95 | 33 | 62 |  |
| LDL (mmol/L) |  |  |  | 0.263 |
| ≤3.12 | 161 | 51 | 110 |  |
| > 3.12 | 36 | 8 | 28 |  |
| ALP (U/L) |  |  |  | 0.152 |
| ≤90 | 150 | 41 | 109 |  |
| > 90 | 47 | 18 | 29 |  |
| Creatinine (mg/dL) |  |  |  | 0.015 |
| ≤0.916 | 79 | 16 | 63 |  |
| > 0.916 | 118 | 43 | 75 |  |
| Uric acid (μmol/L) |  |  |  | <0.001 |
| Normal | 116 | 21 | 95 |  |
| Abnormal | 81 | 38 | 43 |  |
| Urine pH |  |  |  | 0.741 |
| < 6 | 40 | 13 | 27 |  |
| 6 | 115 | 32 | 83 |  |
| > 6 | 41 | 14 | 28 |  |
| Urine crystal |  |  |  | 0.839 |
| Positive | 22 | 7 | 15 |  |
| Negative | 175 | 52 | 123 |  |
| Bilateral |  |  |  | <0.001 |
| Yes | 88 | 43 | 45 |  |
| No | 109 | 16 | 93 |  |
| Multiple |  |  |  | <0.001 |
| Yes | 125 | 56 | 69 |  |
| No | 72 | 3 | 69 |  |
| Stone composition |  |  |  | 0.316 |
| Calcium oxalate | 121 | 33 | 88 |  |
| Carbonate apatite | 50 | 15 | 35 |  |
| Others | 26 | 11 | 15 |  |
| Intervention |  |  |  | 0.011 |
| ESWL | 24 | 5 | 19 |  |
| PCNL | 88 | 36 | 52 |  |
| RIRS | 85 | 18 | 67 |  |

BMI, body mass index; PTH, parathyroid hormone; HDL, high density lipoprotein; LDL, low density lipoprotein; ALP, alkaline phosphatase; ESWL, extracorporeal shock wave lithotripsy; PCNL, percutaneous nephrolithotomy; RIRS, retrograde intrarenal surgery.
